# The Efficacy of the EUS for the Detection of Recurrent Disease in the Anastomosis of Colon

**DOI:** 10.1155/DTE.7.149

**Published:** 2001

**Authors:** Shunichi Nakajima

**Affiliations:** Third Department of Internal Medicine Toho University School of Medicine 2-17-6 Ohashi Meguro-Ku Tokyo 153-8515 Japan

## Abstract

Patients who underwent surgical resection of an advanced colorectal cancer during the period from June 1982 to July 2001 were examined for evidence of no anastomotic recurrence or recurrent lesions through combination of endoscopic ultrasonography (EUS) with endoscopy. Included in this study were 11 patients with recurrence and 36 patients without recurrence, 47 patients in all. Endoscopy revealed stenosis in 81.8% of patients with ana anastomotic recurrence, erosion including cancer exposure in 81.8% and submucosal tumor-like elevation in 45.5%. In the group of patients without recurrence it revealed stenosis in 13.9% of patients, erosion in 22.2%, and a scar-like change in 77.8%. There was a significant difference between the two groups in each change. EUS, on the other hand, revealed localized hypertrophy of the region extending from the submucosa to the mp due to edema early in the postoperative course. The rate of definitive diagnosis with EUS was 100%, compared to 90.1% for endoscopy. The results of this study indicate that EUS is helpful in detecting anastomotic recurrence of colorectal cancer.


*Diagnostic and Therapeutic Endoscopy*, Vol. 7, pp. 149–158
Reprints available directly from the publisher
Photocopying permitted by license only

© 2001 OPA (Overseas Publishers Association) N.V.
Published by license under
the Harwood Academic Publishers imprint,
part of Gordon and Breach Publishing
a member of the Taylor & Francis Group.
All rights reserved.

### The Efficacy of the EUS for the Detection of Recurrent Disease in the Anastomosis of Colon

SHUNICHI NAKAJIMA*

*Third Department of Internal Medicine, Toho University School of Medicine, 2-17-6 Ohashi Meguro-Ku, Tokyo 153-8515, Japan*

*(Received 6 June 2001; Revised 3 October 2001; In final form 22 October 2001)*

Patients who underwent surgical resection of an advanced colorectal cancer during the period from June 1982 to July 2001 were examined for evidence of no anastomotic recurrence or recurrent lesions through combination of endoscopic ultrasonography (EUS) with endoscopy. Included in this study were 11 patients with recurrence and 36 patients without recurrence, 47 patients in all. Endoscopy revealed stenosis in 81.8% of patients with anastomotic recurrence, erosion including cancer exposure in 81.8% and submucosal tumor-like elevation in 45.5%. In the group of patients without recurrence it revealed stenosis in 13.9% of patients, erosion in 22.2%, and a scar-like change in 77.8%. There was a significant difference between the two groups in each change. EUS, on the other hand, revealed localized hypertrophy of the region extending from the muscularis propria (mp) to the serosa in 63.6% of patients with recurrence. In the group of patients without recurrence, and an extramural tumor in 36.4%. EUS revealed hypertrophy of the region extending from the submucosa to the mp due to edema early in the postoperative course. The rate of definitive diagnosis with EUS was 100%, compared to 90.1% for endoscopy. The results of this study indicate that EUS is helpful in detecting anastomotic recurrence of colorectal cancer.

*Keywords*: Anastomotic recurrence; Colorectal cancer; Endoscopic ultrasonography; Postoperative recurrence

### INTRODUCTION

Postoperative recurrence of an advanced colorectal cancer is a serious threat to both patient and physician, and there is complete unanimity of opinion as to the necessity of strict observation after surgery. It is important that anastomotic recurrence in particular be detected and treated early, since it materially affects the outcome. The morphology of the postoperative colonic anastomosis differs from the early postoperative course within one year to the stage of over one year, but it carries a risk of anastomotic

*Tel.: +81-3-3468-1251. Fax: +81-3-3468-1269. E-mail: snaka@t3.rim.or.jp

TABLE I Comparison of clinical and histological characteristics in each group

| | Local recurrence ($n$ = 11) | Non-local recurrence ($n$ = 36) | |
|---|---|---|---|
| Gender | | | |
| Male | 6 | 22 | N.S. |
| Female | 5 | 14 | |
| Age | 63.4 | 62.8 | |
| Location | | | |
| R | 5 | 9 | N.S. |
| S | 4 | 18 | |
| D | 0 | 1 | |
| T | 2 | 4 | |
| A | 0 | 3 | |
| C | 0 | 1 | |
| Depth of tumor invasion | | | |
| mp | 0 | 6 | N.S. |
| s ~ ss | 11 | 30 | |
| Histological type | | | |
| Well differentiated | 8 | 29 | N.S. |
| Moderately differentiated | 3 | 6 | |
| Poorly differentiated | 0 | 1 | |
| Lymphatic invasion | | | |
| ly0 or ly1 | 1 | 9 | N.S. |
| ly2 or ly3 | 10 | 27 | |
| Lymph node metastasis | | | |
| Positive | 7 | 20 | N.S. |
| Negative | 4 | 16 | |
| Stage (TNM) | | | |
| I | 0 | 6 | N.S. |
| II | 10 | 26 | |
| III | 1 | 4 | |

R: rectum, S: sigmoid colon, D: descending colon, T: tranceverse colon, A: ascending colon, C: cecum.

recurrence, whichever the stage may be. In this study, the usefulness of endoscopy and endoscopic ultrasonography (EUS) in the detection of recurrence was evaluated by observing the colonic anastomosis after surgical resection of a cancer and determining the type of anastomotic recurrence.

### MATERIAL AND METHODS

Forty-seven patients who underwent curative surgical resection of an advanced colorectal cancer during the period from June 1982 to July 2001 were examined in this study. They consisted of 11 patients with recurrence and 36 patients without recurrence. Six of the patients with recurrence were males and 5 females, with a mean age of 63.4 years. Twenty-two of the patients without recurrence were males and 14 females, with a mean age of 62.8 years. The site of primary growth in the group with recurrence was the rectum in 5 patients, the sigmoid colon in 4, and the transverse colon in 2. In the group without recurrence, the site of primary growth was the rectum in 9 patients, sigmoid colon in 18, the descending colon in 1, the transverse colon in 4, the ascending colon in 3 and the cecum in 1. The localization of lesions was not taken into consideration because there was no difference between endoscopy and EUS in the localization of recurrent lesions.

The depth of invasion. In all patients with recurrence the degree of tumor spread was beyond the serosa. Of the patients without recurrence, 6 had muscularis propria (mp) cancer, and the degree of tumor spread was beyond the serosa. The primary growth in the group with anastomotic recurrence was classified as well differentiated in 8 patients and moderately differentiated in 3 patients; lymphatic permeation ly0 or l were in 1 patient and ly2 or 3 in 10

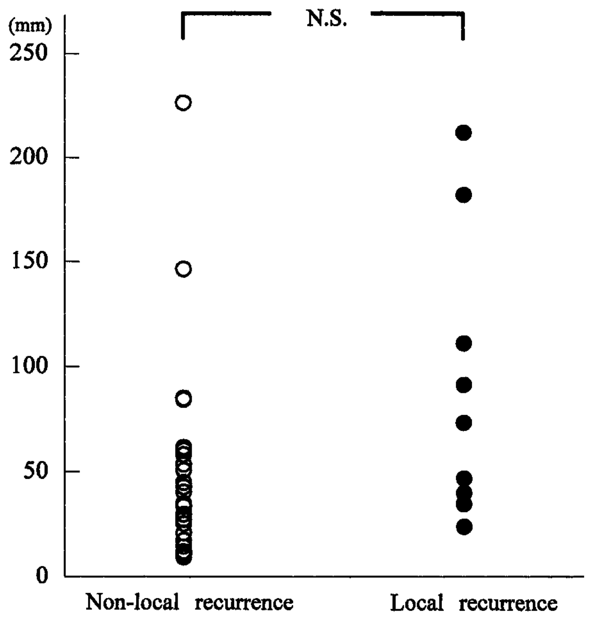

FIGURE 1 Surgical margin from the tumor.

patients; 7 patients were positive for lymph node metastasis and 4 were negative. In the group without recurrence, the primary growth was classified as well differentiated in 29 patients, moderately differentiated in 6 patients, and poorly differentiated in 1 patient; lymphatic permeation ly0 or 1 were in 9 patients and ly2 or 3 in 27 patients; 20 patients were positive for lymph node metastasis and 16 were negative. According to the TNM classification, the group of anastomotic recurrence revealed stage II in 10 patients and stage III in 1 patient and in the group without recurrence, stage I in 6, II in 26 and III in 4 patients (Table I). There was no difference in the distribution of histologic factors between the 2 groups. There was again no difference between mp cancer and cancers invading beyond the serosa with respect to type of recurrence.

The background factors of cancer were investigated, and the state of the anastomosis was examined with endoscopy and EUS. The items of investigation were (1) distance from the surgical material to the stump formed after resection of the primary growth, (2) endoscopic findings of the anastomosis, (3) EUS findings of the anastomosis, and (4) rates of accurate diagnosis by endoscopy and EUS.

The instruments used in this study were colonoscopes (CF-200I, CF-230I, and CF-Q240I of Olympus Optical Company, Tokyo, Japan), an ultrasonographic scope (CF-UM200, 7.5 or 12 MHz), thinner sonographic US probes (UM-2R, 12 MHz, UM-3R, 20 MHz, UM-3D2R, 12 MHz, and UM-3D3R, 20 MHz), and EUS observation systems (EU-IP2, UM-30, and EU-M30). A probe must be selected according to the obtained image. If the image was not visualized perfectly by deep attenuation, probe was exchanged to low frequency one and the lesions and surrounding tissues filled with deairated water were observed in all patients. The results of observation were assessed with the Mann–Whitney test and $\chi^2$-test for the significance of difference. A $p$ value of less than 0.05 was considered to be statistically significant.

### RESULTS

### Distance to the Stump (Fig. 1)

Since there may be cases where cancer cells are remaining at the stump from surgical resection of the lesion for cause of recurrence, the distance from the surgical material to the stump was compared between the 2 groups. Whereas the distance ranged from 15 to 215 mm (average 83.9 mm) in the group without recurrence, it ranged from 8 to 225 mm (average 55.5 mm) in the group with recurrence. The average distance was shorter in the group with recurrence, but there was no significant differences in distance between the 2 groups.

### Endoscopic Findings of the Anastomosis

The anastomosis was examined periodically with the colonoscope. The endoscopic findings of the anastomosis varied with time. Early in the postoperative course (2 months after surgery) edema, redness, and erosion were observed at the anastomosis (Fig. 2a). Six months or more after surgery, slight stenosis and scar formation was observed, but the erosion and edema were no longer present (Fig. 2c). This state was considered to represent a stage of disappearance of operative reaction. On the

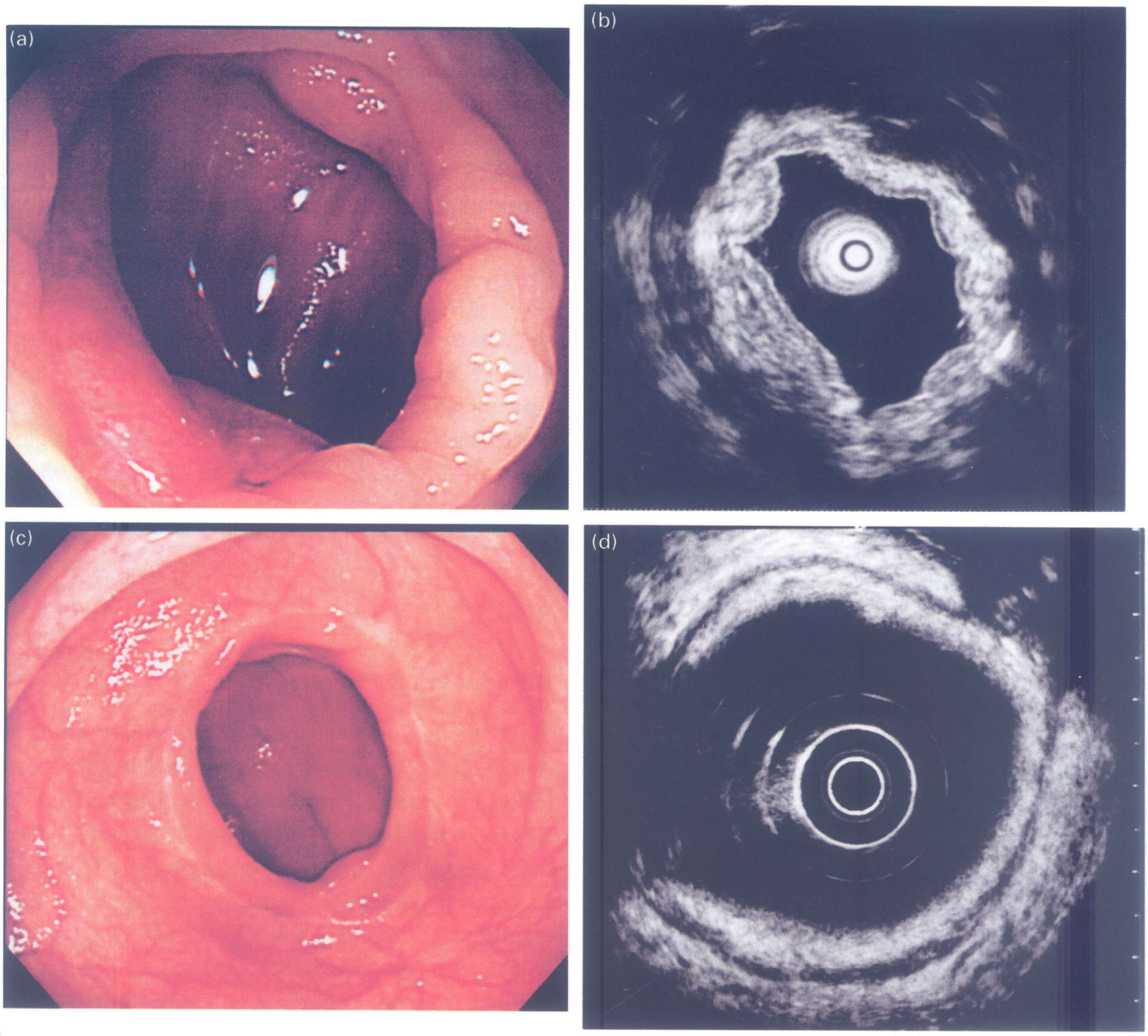

FIGURE 2 Image of post operative phase without recurrence: (a) an endoscopic image with erosion and edem and (b) EUS image with even thickness of third and fourth layer was demonstrated of early postoperative phase within one year; (c) an endoscopic image and (d) EUS image with even thickness of fourth layer was demonstrated of late phase over one year.

other hand, luminal stenosis and friability, unilateral, irregular, coarse mucosa exposing a cancer was observed in some patients (Fig. 4a).

Endoscopic findings were compared between the group of patients without recurrence and the group with recurrence. The endoscopic findings were broadly classified as follows: difficult navigation as stenosis, mucosal erosion including invasion by the tumor, submucosal tumor (SMT)-like elevation, and a scar-like change.

In the group with recurrence, stenosis was observed in 9 patients (81.8%), erosion including cancer exposure in 9 (81.8%), SMT-like elevation in 5 (45.5%), and scar-like change in 0 (0%). In the group without recurrence, on the other hand, stenosis was observed in 5 patients (13.9%), erosion in 8 (22.2%), SMT-like elevation in 0 (0%), and scar-like change in 28 (77.8%). There was a significant difference between the 2 groups in the frequency of each change (Table II).

TABLE II Endoscopic finding of anastomotic site

| | Stenosis | Erosion | Submucosal elevation | Scar |
|---|---|---|---|---|
| Local recurrence ($n = 11$) | 9 (81.8%)* | 9 (81.8%)* | 5 (45.5%)* | 0 (0%)* |
| Non-local recurrence ($n = 36$) | 5 (13.9%)* | 8 (22.2%)* | 0 (0%)* | 28 (77.8%)* |

$*p < 0.01$.

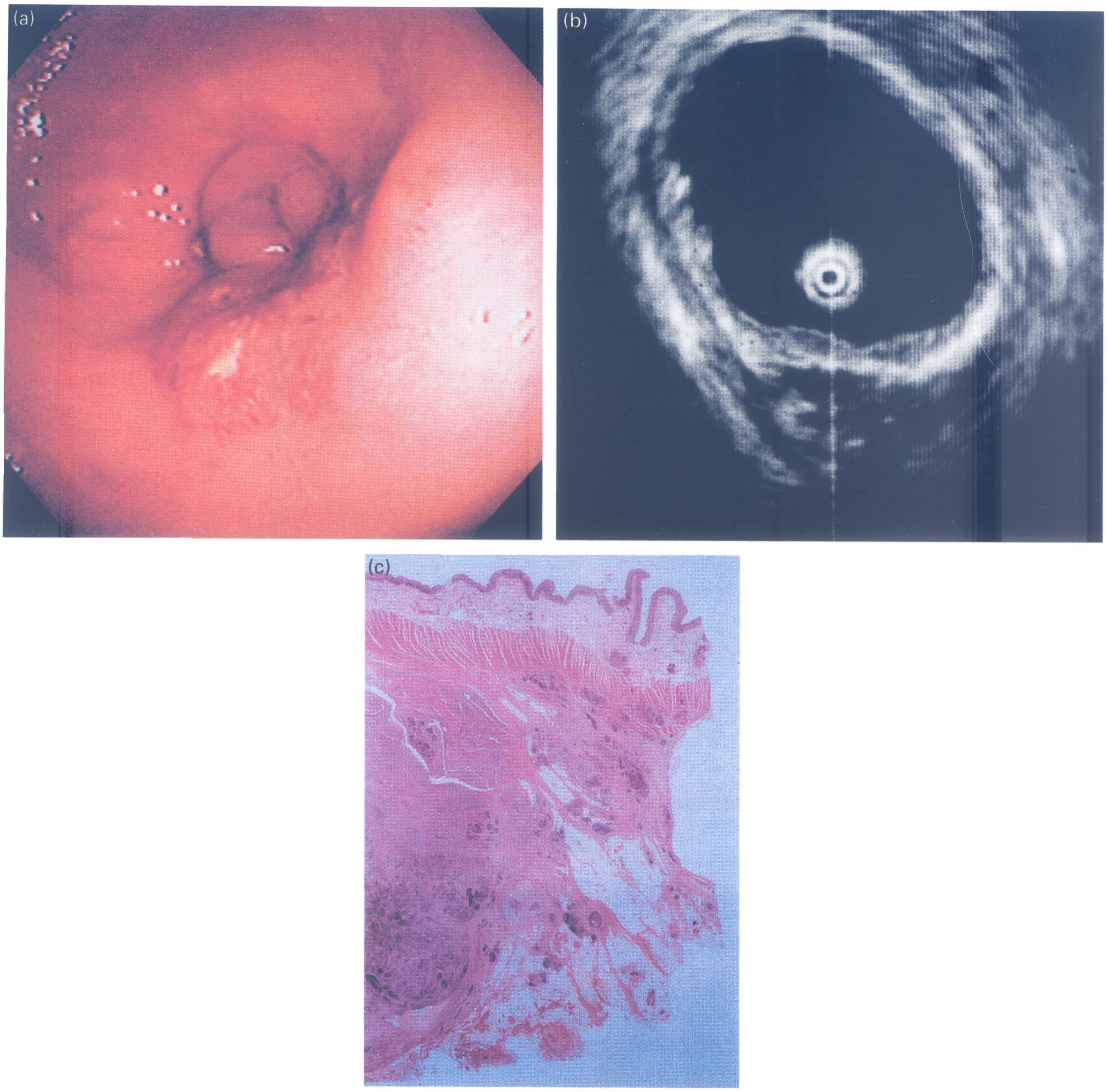

FIGURE 3 Image of early recurred case within one year: (a) an endoscopic image with erosion and submucosal elevation; (b) EUS image with localize hypertrophy of fourth layer was imaged; (c) pathological section revealed massive tumor mass in the extra colonic part.

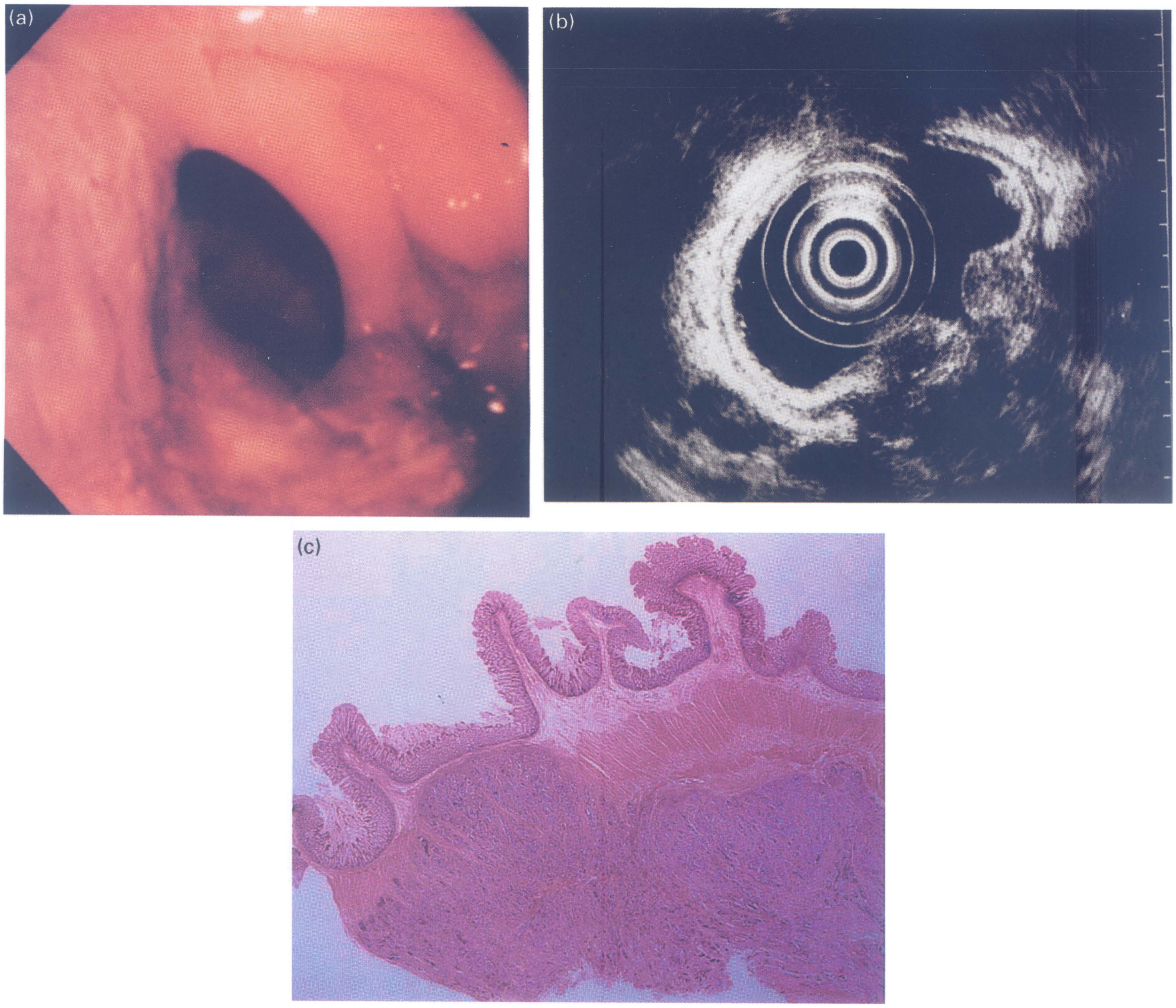

FIGURE 4 Image of late recurred case over one year: (a) an endoscopic image with erosion and stenosis; (b) EUS image with heterogeneous mass; (c) pathological section revealed massive tumor mass in the extra colonic part.

### EUS Findings of the Anastomosis

Following endoscopic observation the anastomosis filled with deaerated water was observed with EUS.

### *Without Recurrence*

The third layer corresponding to the submucosa and the fourth layer mp showed circumferential hypertrophy due to surgery (Fig. 2b). The hypertrophy of the submucosa disappeared with time, and the mp of circumferentially nearly even thickness was visualized (Fig. 2d). The anastomosis showed hypertrophy of the mp, compared to normal condition, but the mp was imaged circumferentially even thickness. In the stage of disappearance operative reaction after surgery the mp alone showed circumferential hypertrophy.

### *With Recurrence*

Intense stenosis, erosion due to cancer exposure, and SMT-like elevation (Fig. 3a) were observed at the anastomosis. The fourth layer of about 1/4 of the

TABLE III Various thickness of fourth layer in EUS

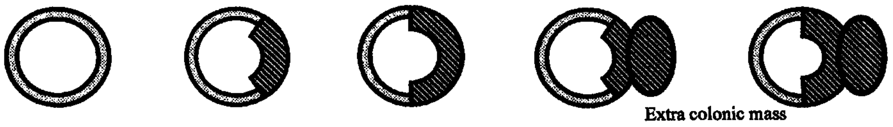

| | | | | | |
|---|---|---|---|---|---|
| Local recurrence ($n = 11$) | 0% ($n = 0$) | 27.2% ($n = 3$) | 36.4% ($n = 4$) | 18.2% ($n = 2$) | 18.2% ($n = 2$) |
| Non-local recurrence ($n = 36$) | 100% ($n = 36$) | 0% ($n = 0$) | 0% ($n = 0$) | 0% ($n = 0$) | 0% ($n = 0$) |

$p < 0.05$

monotonous thickening of mp layer.

tumor image.

intestinal circumference was thickened on EUS images, and high and low echoes were mixed in the affected region (Fig. 3b). Consistent with the anastomosis, the histologic section of resected tissue showed recurrence on the side of the serosa continuous with the mp (Fig. 3c).

When the tumor recurred one year or more after surgery, the mucosal structure was destroyed unilaterally, and an extramural tumor was demonstrated (Fig. 4b). Consistent with EUS images, the tissue specimen obtained at surgery showed the focus of extramural recurrence (Fig. 4c).

The EUS images of the anastomosis were classified into varying grades of change. Even circumferential hypertrophy of the fourth layer (mp) at the stage of disappearance of operative reaction was observed in all patients. Local hypertrophy of the fourth layer was classified into less than 25%, 25–50%, 50–100%, and an extramural tumor (Table III). In the group of patients with recurrence, even hypertrophy of the fourth layer alone was observed in 0 patient (0%). Mural hypertrophy due to less than 25% hypertrophy due to recurrence in 3 patients (27.2%), tumor-free 25–50% mural hypertrophy due to recurrence in 4 patients (36.4%). Less than 25% mural hypertrophy with an extramural tumor due to recurrence in 2 patients (18.2%) and 25–50% mural hypertrophy with an extramural tumor due to recurrence in 2 patients (18.2%). But even hypertrophy of the fourth layer was visualized in all patients without recurrence. There were obvious differences between the 2 groups in EUS findings.

EUS images were compared between patients with early recurrence (within one year) and patients with late recurrence (over one year) (Table IV). Whereas less than 25% hypertrophy of the region extending from the mp to the serosa was observed in 66.7% of patients with early recurrence, half of circumferential hypertrophy in 62.5% of patients with late recurrence. An extramural tumor was observed in 0% with early

TABLE IV EUS finding of recurred carcinoma

| | Circumferential hypertrophy of fourth to fifth layer (%) | | | Extra-colonic mass (%) | |
|---|---|---|---|---|---|
| | Less than 25% | 25–50% | More than 50% | Negative | Positive |
| Early recurrence (within one year) ($n = 3$) | 66.7 | 33.3 | 0 | 100 | 0 |
| Last recurrence (over one year) ($n = 8$) | 37.5 | 62.5 | 0 | 50 | 50 |

TABLE V Diagnostic rate of recurred carcinoma in each procedure

| | | Endoscopy (%) | EUS (%) |
|---|---|---|---|
| Local recurrence | Definite | 90.1 | 100 |
| ($n = 11$) | Suspicious | 9.9 | 0 |
| | Impossible | 0 | 0 |
| Non-local recurrence | Definite | 97.1 | 100 |
| ($n = 36$) | Suspicious | 2.9 | 0 |
| | Impossible | 0 | 0 |

recurrence, while it was demonstrated in 50% with late recurrence. The later signs of recurrence were found, the greater the degree of circumferential involvement, the higher was the frequency of an extramural tumor.

### *Rate of Diagnosis*

Patients with advanced colorectal cancer are often left with erosion and an ulcer early in the postoperative course and, therefore, may be suspected of having recurrence by endoscopy. A diagnosis of recurrence could not be made in all patients with late recurrence because of flexure or adhesion. Anastomotic recurrence could be diagnosed by endoscopy alone in 90.1% of patients (Table V), but no definitive diagnosis could be made in 1 patient (9.9%) because of poor distension of the colon. Furthermore, recurrence could not be ruled out in 1 (2.9%) of the patients without recurrence because of erosion and edema occurring early in the postoperative course. With endoscopy combined with EUS, on the other hand, the rate of diagnosis was 100%.

### DISCUSSION

Advanced colorectal cancer is known to recur in the anastomosis or the pelvic cavity. Recurrence in the anastomosis can arise from the implantation of cancer cells into the pelvic cavity or the stump or recurrence in the stump. Anastomotic recurrence can arise from the lymph nodes left with cancer cells, the surface of ablation or the pelvic cavity implanted with cancer cells. Implantation of cancer cells has been demonstrated in animal studies [1], but it is not yet to be elucidated in detail in humans [2]. Distance metastases are detected by abdominal and thoracic computed tomography (CT) and determination of tumor markers, such as CEA, while local recurrence is detected by the barium enema examination and pelvic CT or magnetic resonance imaging (MRI). However, since differentiation of recurrence from fibrotic changes by pelvic CT is difficult depending on the case, this technique is not adequate for early detection [3]. As a matter of fact, the abdominal CT scan obtained 6 months after surgery was not remarkable in one of our patients in whom recurrence was detected by change by endoscopy 8 months after surgery. When the patient was operated on again at a later date, the tumor was found to have invaded beyond the mucosa superior to the colon into the serosa. The reason for failure to detect recurrence by CT seems that the lesion of recurrence in the pelvic cavity escaped detection because examination frequently performed around the liver. However, it seems undesirable to repeat the CT scan extensively because it involves exposure to radiation.

MRI provides for qualitative diagnosis of fibrous changes and lesions of recurrence by comparing the relaxation time between lesion of recurrence and fibrous tissue [4,5]. However, since the MRI scan has limitations in the study of the intestinal tract with peristalsis, in some of the patients the lesion of recurrence was demonstrated by MRI after detection by endoscopy, but the tumor was rather advanced in the pelvic cavity. Furthermore, MRI was not performed periodically on all the patients. Therefore, its usefulness cannot be affirmed.

The rate of recurrence at the anastomosis has been reported to range from 0.8 to 1.4% for colonic cancer [6,7] and from 7.8 to 13% for the rectal cancer [8–10]. The time to recurrence is two years or less in most cases [11,12]. Such was the case in 9 of 11 patients in this study.

As for the relationship of demographic characteristics of patients with recurrence, there was no sex difference, but the age tended to be lower for the group of patients with recurrence.

However, the tumor is often well advanced by the time the lesion of recurrence is detected. It can be operated on again, but the prognosis is by no means favorable. This is because the lesion of anastomotic recurrence is difficult to detect in early post-operative period. The tumor is not visualized unless grown to a certain size. In anastomotic recurrence, in particular the tumor grows in the intestinal wall and on the serosal side. The lesion of anastomotic recurrence is already well advanced at the time an abnormality is detected on the mucosal side by colonoscopy.

Postoperative endoscopy is performed periodically to examine the anastomosis and detect a new lesion in the remnant intestine, but the state of the anastomosis differs from the early postoperative stage to the later stage of stabilization. Erosion, an ulcer, and an edematous change are observed on the mucosa until about 6 months after surgery, and these changes become scarred over time so that it is difficult to identify the anastomosis in not a few instance [13–15]. In some of the patients with erosion, redness, and stenosis were revealed by endoscopy early in the postoperative course and only a scar was observed by re-examination several months later. In some cases, however, it is difficult to distinguish an early change in the anastomosis from recurrence. In cases where the main lesion is in the submucosa and is not exposed on the mucosa, cancer cells are difficult to be detected even by biopsy.

Endosonography was introduced into the observation of the submucosal condition in the late 1980s, and attempts have since been made to detect anastomotic recurrence after surgery for colorectal cancer [16–20]. In the early days of intrarectal sonography, a probe was passed blindly through the rectum because this technology was not provided with endoscopic function. Therefore, only the rectum and neighboring region could be observed by intrarectal sonography. Around 1990 a dedicated instrument with endoscopic function was introduced [21,22] so that the entire colon could be examined by EUS. Furthermore, a thin ultrasonic probe came into use so that an ultrasonic diagnosis could be made if the probe negotiated the stenosis, no matter how severe it was [23]. The thin ultrasonic probe came into widespread clinical use, [24,25] and the frequency became variable with EUS. As a result, it became possible to make an ultrasonic diagnosis with judicious combination of thinner probes of different frequency. Furthermore, it has been reported that lymph nodes neighboring the serosa can be biopsied under ultrasonic guidance using the fine needle [26–28].

EUS has been performed at our center to detect colorectal cancers since 1989, and its usefulness in the evaluation of the depth of invasion has been established to a certain extent [22]. For the examination of the anastomosis without recurrence, high frequency probe (12–20 MHz) can be used and more low frequency probe (7.5 MHz) must be used for the purpose of detecting deep-lying lesion of recurrence because of deep attenuation.

Circumferential hypertrophy of the third and fourth layer of the colon due to submocosal edema were demonstrated until about 6 months after surgery, but hypertrophy of the third layer was not longer observed in the stage of disappearance of operative reaction. However, circumferential hypertrophy of the fourth layer, the mp, was visualized even seven years after surgery. This finding suggests that the local change in the mp can serve as an indicator of postoperative anastomotic recurrence. In other words, it is suggested that even hypertrophy of the fourth layer can be regarded as the absence of anastomotic recurrence and that localized hypertrophy is a sign of recurrence. At present, however, EUS is not performed on all patients to follow their postoperative course, and it is generally followed by endosocopy or the barium enema examination. Periodic observation was not conducted or the observation interval was widely spaced in almost all patients with local recurrence in this study so that EUS did not lead to early detection of recurrence. It seems desirable that EUS and endoscopy should be performed in combination at intervals of about 3 months within two years after surgery in the high-risk group of recurrence. Furthermore, EUS provides reliable information about the extra- and intramural condition. It is very useful in this aspect and should therefore be performed aggressively in the patient with high risk of recurrence.

### CONCLUSION

Postoperative anastomotic recurrence of colorectal cancer could be diagnosed by endoscopy at a high rate, but the combination of endoscopy with EUS seems essential for definitive diagnosis of anastomotic recurrence of advanced colorectal cancer.

### *Acknowledgements*

We would like to offer our sincerest thanks to Professor Yoshihiro Sakai for assisting in completion of the manuscript of providing guidance and feedback, and Dr Kazuya Yoshimoto for his guidance throughout the study. We would also like to thank the medical staff of the Third Department of Internal Medicine at Toho University School of Medicine for their assistance in preparing the specimens.

### *References*

[1] Matsumoto, M., Maruta, M., Maeda, K., Utsumi, T., Tohyama, K. and Masumori, K. (1999) "Suture line recurrence following curative resection for carcinoma of the colon-report of two cases", *J. Jpn Surg. Assoc.* **60**(5), 1341–1344.

[2] Cho, H. (1997) "Suture-line implantation in colorectal cancer surgery and efficacy of providone-iodine solution", *Jpn J. Gastroenterol. Surg.* **30**(8), 1847–1855.

[3] Kelvin, F. (1983) "The pelvis after surgery for rectal carcinoma, serial CT observation with emphasis on non-nepotistic features", *AJR* **140**, 959–964.

[4] Gomberg, J., Friedman, A. and Radecki, P. (1986) "MRI differentiation of recurrent colorectal carcinoma from post-operative fibrosis", *Gastrointest. Radional.* **11**, 361–363.

[5] Gabriel, P., Wolfgang, S. and Gerd, F. (1988) "Recurrent rectal caner: diagnosis with MR imaging versus CT", *Radiology* **168**, 307–311.

[6] Tsunoda, A., Kawamura, M. and Nakao, K. (1993) "Recurrence at the suture line following resection for carcinoma of the colon", *J. Jpn Soc. Colo-Proctol.* **46**, 215–218.

[7] Hardy, K.J., Cuthbertson, A.M. and Hughes, E.S.R. (1971) "Sutere-line neoplastic recurrence following large-bowel resection", *Aust. NZ J. Surg.* **41**, 44–46.

[8] Takasaka, H., Sasaki, K., Yamashiro, K., Yamaguchi, H., Tutai, K., Okada, Y., Mizuguchi, T. and Hirata, K. (1995) "Studies on local recurrence of rectal cancer and its surgical treatment", *Hokkaido J. Surg.* **40**, 126–129.

[9] Lee, Y. (1996) "Local and regional recurrence of carcinoma of the colon and rectum", *Surg. Oncol.* **5**, 1–13.

[10] Nicola, P., Leopoldo, S., Renato, C., Ouchemi, C., Grattarola, M. and Peracchia, A. (1998) "Role of follow-up in management of local recurrences of colorectal cancer", *Dis. Col. Rect.* **41**(9), 1127–1133.

[11] Delpero, J.R., Bardou, V., Moutardier, V., Hardwigsen, J., Granger, F. and Houvenaeghel, G. (1998) "Surgical resection of locally recurent colorectal adenocarcinoma", *Br. J. Surg.* **85**, 372–376.

[12] Eroglu, A., Sever, N., Altinok, M. and Yavuz, Y. (1997) "Risk factor associated with local recurrence after curative for rectal cancer", *Surg. Today Jpn J. Surg.* **27**, 1113–1118.

[13] Kawaguchi, T., Katagiri, K., Kishi, H. and Sakai, Y. (1990) "Study on endoscopic findings after colectomy", *Prog. Digest. Endosc.* **36**, 132–135.

[14] Yasuda, M., Kawaguchi, T. and Sakai, Y. (1992) "Endoscopic technique for the colon after surgical treatment", *Gastro-intestest. Endosc.* **4**(11), 1579–1583.

[15] Nakajima, H. (1992) "Endoscopic follow up and findings after resection of colorectal cancer", *Gastrointestest. Endosc.* **4**(11), 1625–1631.

[16] Beynon, J., Mortensen, N.J., Foy, D.M., Channer, J.L., Rigby, H. and Virjee, J. (1989) "The detection and evaluation of locally recurrent rectal cancer with rectal endosonography", *Dis. Col. Rect.* **36**(3), 509–517.

[17] Mascagni, D., Corbellini, L., Urciuoli, P. and Matteo, G.Di (1989) "Endoluminal ultrasound for early detection of local recurrence of rectal cancer", *Br. J. Surg.* **76**(11), 1176–1180.

[18] Dresing, K. and Stock, W. (1990) "Ultrasonic endoluminal examination in the follow up of colorectal cancer", *Int. J. Colorect. Dis.* **5**, 188–194.

[19] Tschmelitsch, J., Glaser, K., Schwarz, C., Judmair, G. and Bodner, E. (1992) "Endosonography in the diagnosis of recurrent cancer of the rectum", *J. Ultrasound Med.* **11**, 149–153.

[20] Romano, G., Esercizio, L., Santangelo, M., Vallone, G. and Santangelo, M. (1993) "Impact of computed tomography vs. intrarectal ultrasound on the diagnosis, resectability, and prognosis of locally recurrent rectal cancer", *Dis. Col. Rect.* **36**(3), 261–265.

[21] Katsumata, K., Kimura, K., Koyanagi, Y., Tani, C., Nakajima, A., Kato, K., Kubouchi, T. and Sumi, T. (1993) "Endoscopic ultrasonography for recurrence at the anastomosed site following low anterior resection for rectal cancer", *J. Jpn Pract. Surg. Soc.* **54**(9), 2324–2328.

[22] Yoshimoto, K., Takada, H. and Sakai, Y. (1991) "The evaluation and prospect on the endoscopic ultrasonography for colorectal cancers", *Prog. Digest. Endosc.* **44**, 54–58.

[23] Kakemura, T., Inoue, H., Kobayashi, H. and Sakai, Y. (1994) "Evaluation of small-caliber ultrasonic probes in the clinical diagnosis of colorectal lesions", *Prog. Digest. Endosc.* **45**, 83–87.

[24] Kaneko, Y., Tashiro, H., Sasaya, K., Murata, S., Iwamoto, K., Miura, E., Ando, H. and Itsubo, K. (1992) "A case report of significance of endoscopic ultrasonography for an outside-wall recurrent case of colon cancer", *Prog. Digest. Endosc.* **41**, 345–348.

[25] Lightdate, C.J. (1995) "Detection of anastomotic recurrence by endoscopic ultrasonography", *Gastrointest. Endosc.* **5**(3), 595–600.

[26] Nielsen, M., Pedersen, J., Hald, J. and Christiansen, J. (1992) "Recurrent extraluminal rectal carcinoma: transrectal biopsy under sonographic guidance", *AJR* **158**, 1025–1027.

[27] Giovannini, M., Bernardini, D., Seitz, J.F., Moutardier, V., Hoevenaeghel, G., Monges, G. and Delpero, J.R. (1998) "Value of endoscopic ultrasonography for assessment of patients presenting elevated tumor marker levels after surgery for colorectal cancer", *Endoscophy* **30**, 469–476.

[28] Woodward, T. and Menke, D. (2000) "Diagnosis of recurrent rectal carcinoma by EUS-guided fine-needle aspiration", *Gastrointestest. Endosc.* **51**(2), 223–225.

